# A comparison of skin dose estimation between thermoluminescent dosimeter and treatment planning system in prostatic cancer: A brachytherapy technique

**Published:** 2021-01-27

**Authors:** Mehrsa Majdaeen, Soheila Refahi, Amin Banaei, Mahdieh Ghadimi, Mahdieh Afkhami Ardekani, Nouraddin Abdi Goushbolagh, Hamed Zamani

**Affiliations:** ^1^Department of Radiotherapy and Oncology, Razi Hospital, Guilan University of Medical Sciences, Rasht, Iran; ^2^Department of Medical Physics, Faculty of Medicine, Ardabil University of Medical Sciences, Ardabil, Iran; ^3^Department of Medical Physics, Faculty of Medical Sciences, Tarbiat Modares University, Tehran, Iran; ^4^Department of Medical Physics, Faculty of Medicine, Shahid Sadoughi University of Medical Sciences, Yazd, Iran; ^5^Department of Radiology, Faculty of Para-Medicine, Hormozgan University of Medical Sciences, Bandar-Abbas, Iran

**Keywords:** Brachytherapy, prostatic malignancy, thermoluminescent dosimeters, treatment planning system

## Abstract

**Aims:**

This study aimed to compare the skin dose calculated by treatment planning system (TPS) and measured with thermoluminescent dosimeters (TLDs) in brachytherapy of prostatic cancer to show the skin TLD dosimetry as an appropriate quality assurance procedure for TPS dose calculations.

**Methods:**

The skin dose of 15 patients with prostatic cancer treated by high dose rate brachytherapy technique was assessed by two types of TLD dosimeters (GR-200 and TLD-100). The TLDs were placed on the patient’s skin at three different points (anterior, left, and right) using five TLDs for each point. The dose values of TLDs and TPS were compared using paired t-test and the percentages of difference were reported.

**Results:**

There was a good agreement between TPS calculations and TLDs measurements for both of the GR-200 and TLD-100 dosimeters. The mean skin dose values for anterior, left, and right points were 65.06±21.88, 13.88±4.1, and 10.05±4.39 cGy, respectively, for TPS. These values were 65.70±23.2, 14.51±4.3, and 10.54±5 cGy for GR-200, and 64.22±23.5, 13.43±4.4, and 9.99±4.1 cGy for TLD-100, respectively.

**Conclusion:**

The TPS skin dose calculations in brachytherapy of prostatic cancer had a good agreement with the TLD-100 and GR-200 measurements at the three different points on patients’ skin. TLD-100 had lower differences with TPS calculations compared to GR-200.

**Relevance for Patients:**

The outcome of this research shows that for people with prostatic cancer, TPS can estimate accurately the skin dose of different points including anterior, left, and right in brachytherapy technique.

## 1. Introduction

Prostate cancer is the second common cancer in the world among men and constitutes fifth most common cause of death [1]. Very few patients are diagnosed with prostate cancer <50 years and most of them are diagnosed after 65 years. The cumulative risk of prostate cancer ranges between 0.5 and 20% worldwide at the age of 85 years [2].

With regard to the stage of prostate cancer, the choice of treatment method will have a great impact on the patient’s quality of life. Surgery, chemotherapy, radiotherapy, and immunotherapy are the main treatment methods of prostate cancer. In localized prostate tumors, prostatectomy (surgery) and radiotherapy have been similar therapy efficiency [3]. External and internal radiation-therapy are two branches of radiotherapy. Internal radiotherapy or brachytherapy is often utilized for localized prostate cancer which were previously undergoing external radiation-therapy [4].

The major goal of brachytherapy is to locate radioactive sources into the tumor target to deliver the highest radiation dose to cancer cells and lowest one to normal cells [5,6]. The high-dose-rate (HDR) and low-dose-rate (LDR) are two techniques for prostate cancer treatment. LDR source involves the constant implantation of radioactive seeds, ^125^iodine (^125^I) or ^103^palladium (^103^Pd), into the prostate [7]. HDR ^60^Co source consists of cobalt metal which has a uniform distribution and is kept inside the source cylinder [8]. These sources are utilized for the treatment of prostate cancers due to the longer half-life when compared with the more conventional ^192^Ir source [7,9].

To successfully treat the target volume, the high dose of radiation is required; therefore, dosimetry accuracy is an important issue [10]. Modern brachytherapy machines use a treatment planning system (TPS) to calculate dose distribution around the source dwell positions [11]. Accurate dose calculation would result in a more precise treatment; therefore, verification of TPS accuracy is important. Practical and laboratory methods using radiation detectors are utilized to assess the accuracy of TPS [11]. Based on guidelines to evaluate the dose differences between the dose verification software and the TPS, a rate of 5% inaccuracy should be considered.

Several studies based on *in vivo* and skin dose dosimetry have been reported use of ^192^Ir, ^125^I, and ^103^Pd sources for prostate cancer treatment [7,9], while recently many centers are used ^60^Co (~1.25 MeV) source due to the higher energy [12,13]. This study determined to compare the TPS skin dose calculation for ^60^Co brachytherapy of prostate cancer in patients with skin dose measurements using two types of thermoluminescent dosimeters (TLDs), TLD-100, and GR-200, to show the skin TLD dosimetry as an appropriate quality assurance procedure for TPS dose calculations.

## 2. Material and Methods

In the present research, a HDR BEBIG ^60^Co (model Co0.A86) remote after-loading brachytherapy unit (Eckert & Ziegler BEBIG GmbH, Germany), along with computed tomography (CT)-based brachytherapy TPS, were used for HDR treatments. Fifteen male patients with the average age of 54 years (ranged from 37 to 75) having locally advanced prostate cancer referred for HDR brachytherapy participated in our study. Patients were informed of the whole procedure of TLD dosimetry with attaching 15 TLD chips on their skin. Each patient signs the consent for co-operation in the study. Furthermore, this study was approved by the national Ethics Committee and National Research Ethics Board.

Dose measurements were carried out using TLD-100 (Harshaw Company, Thermo Electron Corporation, Reading, UK), made of LiF, Mg and Ti with dimensions of 3.2×3.2×0.9 mm^3^. Also, GR-200 (LiF: Mg, Cu, P circular chips, SDDML, China) with dimensions of 3.2×3.2×0.2 mm^3^ was used for skin dose measurement.

Before the measurements, all dosimeters were annealed in a TLD annealing furnace (1 h at 400°C and 2 h at 100°C). Furthermore, before the readout, they were pre-heated at 100°C for 20 min. All dosimeters were calibrated at Iran Secondary Standard Dosimetry Laboratory.

### 2.1. TLD calibration

**Figure 1:**
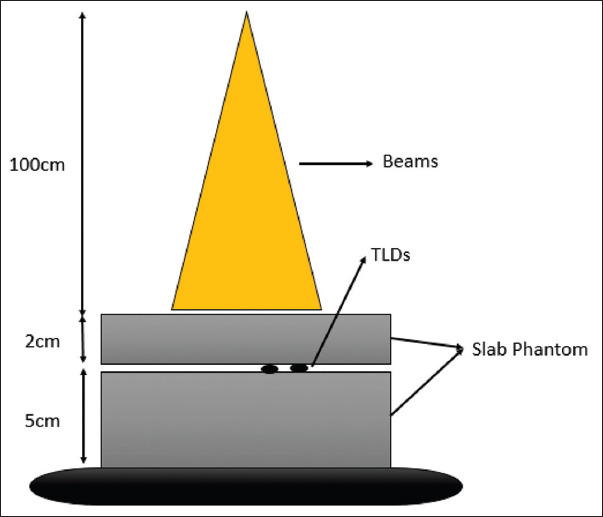
Thermoluminescent dosimeters position to determine the correction factors (C_f_)

For obtaining the calibration curve to estimate the correction factors (C_f_), a multi-layer 30×30 cm^2^ Perspex phantom was designed for irradiation the TLDs. For insertion of the TLDs, a layer with a thickness of 3 mm was created. Twenty holes in the shape of a square (3.2×3.2 mm^2^) and in the form of 4×5 cm^2^ matrix were created in its center. The depth of holes was 2 mm. A layer with a thickness of 2 cm was considered as the depth of treatment. The thickness of 5 cm was considered for the lower back-scatter slab (Figure 1). For irradiation, the TLDs were located into the phantom, and the field size and SSD were chosen 10×10 cm^2^ and 100 cm, respectively. To verify the delivered absolute dose values, a farmer type 30013 ionization chamber (0.6 cc, PTW, Freiburg, Germany) was used at the dosimetric condition similar as the TLD irradiation setup. The phantom was located under the uniform irradiation of a ^60^Co gamma radiation machine (Siemens Gammatron S). Subsequently, all irradiated TLDs were read by LTM reader (Fimel, Velizy, France) after 48 h.

Fifteen TLDs were divided into three groups of five, located in the phantom. Dose levels of 40, 60, 80, 100, and 120 cGy were used to plot the calibration curve. Furthermore, two TLDs were applied to measure the background dose.

### 2.2. Skin dose measurement

The skin dose at the point of interest was calculated using the following equation:

Equation 1: D (TLD) = R×N×G×k

Where R is the corrected TLD reading (in nC), N is the calibration coefficient (in Gy/nC). The correction factor for intrinsic energy dependency was assumed equal to 1 because the calibration energy and brachytherapy sources were ^60^Co. G and k are both correction factors. For all of the measurements, the point of interest was the basal skin layer which is defined at a depth of 0.07 mm beneath of skin surface in accordance with the ICRP and ICRU recommendations [14,15].

In summary, G is the geometry correction factor and accounts for the inverse square relationship between the dose at the point of interest and the point of measurement. Since the point of interest in the skin (0.07 mm) is closer to the source than the measured point (center of the TLD; 0.45mm for TLD-100 and 0.1 mm for GR-200), the source position during the brachytherapy changes in planed dwell positions. Individual G values for each dwell position cannot be calculated and an overall value for G was calculated based on the dwell weights in each position for each patient. It must be noted that increasing the source to skin distance will cause to G has the value near to 1. Our calculated value for G was in the range of 1.0012 to 1.0044 for different TLD positions and patients, which are negligible. The correction factor k accounts the correction factor relates to the lack of electronic equilibrium for skin measurement (without any backscatter material for TLDs). This factor obtained by the Monte Carlo simulation in a previous study [16] with an average value of 0.98, which was applied in our study.

More details of TLD dosimetry for skin in brachytherapy technique have explained by Raffi *et al*. [16] study.

### 2.3. Treatment planning procedure

The procedure of treatments was performed in the following steps. First, the prostate sets with template needles (CT Contour Prostate Template Sets, Eckert & Ziegler BEBIG, Germany) were set on patients body and the needles were positioned in the desired position through the prostate gland in the operating room under the transrectal ultrasound image guide. Aerated gel was inserted into the urinary catheter to visualize the bladder and urethra. Suitable needle composition was suggested by a radiation oncologist according to the tumor staging, geometry, and its margin extension. A pelvic CT scan (3-mm slice thickness) was acquired for treatment planning, and an oncologist delineates HR-CTV, Intermediate CTV (IR-CTV), rectum, sigmoid, and bladder plans for each patient separately. An inverse treatment planning (SagiNova treatment planning software, Version 1.6, Eckert & Ziegler BEBIG, Germany) was used to calculate the positions and time of stoppings (dwell times) of Co-60 source in each position in the needles for obtaining the closest dose distribution to the prescribed dose in the target volume.

The prescribed total dose (external radiotherapy+brachytherapy) for HR-CTV ranges between 80 and 90 Gy EQD2 (equivalent doses delivered in 2-Gy fractions), depending on tumor size in the time of brachytherapy. Table 1 depicts the TPS skin dose calculations for each patient at three different points.

**Table 1:** TPS-based calculated dose (cGy) at three points

Patient number	Anterior	Left	Right
1	120	19	17
2	53	11.2	8.9
3	68.8	13	10
4	79	9	10
5	45.5	13	13
6	62	15	17.9
7	51	9.4	4.9
8	80.1	14	14
9	58	11.5	8.5
10	81	19	5.8
11	64	18.4	15
12	70	16	5
13	75	16.7	7
14	24.5	5	4.8
15	44	18	9

TLD: Thermoluminescent dosimeters, TPS: Treatment planning system

For treatment delivery, the prostate sets with plastic needles (Eckert & Ziegler BEBIG, Germany) were positioned through the guide of ultrasound imaging similar to the procedure performed for CT imaging.

### 2.4. Dosimetry

To measure skin dose, TLD dosimetry (GR-200 and TLD-100) was performed for each patient. The TLDs were placed on the patient’s skin along their symphysis pubis bone (anterior), left and right sides of their pelvic bones. For each point, five dosimeters (five of each TLD model) were placed as close as possible to improve the statistical fluctuation of dosimetry results.

The TLDs and TPS dose differences were calculated following below equation:


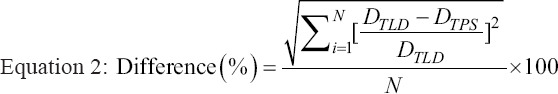


Where D_TPS_ is the delivered dose predicted by TPS, D_TLD_ and N are the absorbed dose of TLD and the number of irradiated groups, respectively.

### 2.5. Uncertainty analysis

An uncertainty analysis was performed to evaluate per-needle and total plan error detection thresholds. Combined relative standard uncertainty (Uc) for the determined dose of TLDs was calculated by equation 3:


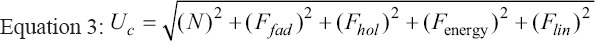


Where N is the calibration coefficient, F_fad_ is the fading correction factor which is equal to 0.02% for TLD-100 and 0.05% for GR-200 according to the Izewska *et al*. [17] study results. In addition, F_hol_ is the TLD holder correction factor and this value is estimated 0.3% and 1% for each energy for TLD-100 and GR-200, respectively [17]. F_energy_ is the energy correction factor measured as the standard error of the actual corrections used for all patients. For each dose, six capsules were irradiated and the dose-response non-linearity correction factor (F_lin_) was determined by making a linear fit to the experimental data.

## 3. Results and Discussion

### 3.1. TLD calibration and uncertainty

**Figure 2:**
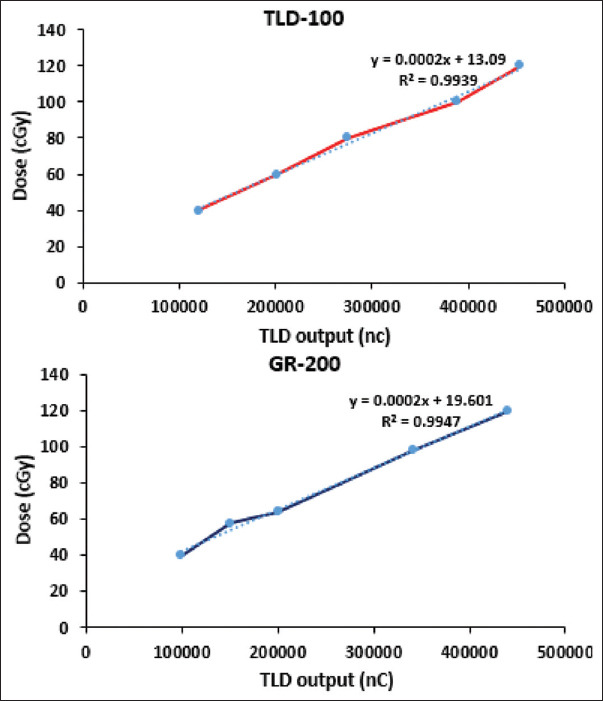
Thermoluminescent dosimeters calibration curves along with calibration coefficient

The obtained TLDs calibration curves are shown in Figure 2. Uncertainty results from different components affected TLD measurements depicted in Table 2. As shown in Figure 1, the calibration coefficient of TLD-100 and GR-200 is 0.0002 at the dose range of 40 – 120 cGy. Furthermore, the R^2^ value of the fitting line was obtained 0.9939 and 0.9947 for TLD-100 and GR-200, respectively. In a study, Altaf *et al*. [18] measured the calibration coefficient of GR-200 in different energies. It was reported that, the calibration coefficient was 4.43, 4.44, 4.03, 4.36, and 4.3, respectively, in energy range from 50 to 150 kV. The discrepancies with the current research are related to the different energy ranges and also doses (which was 0.7 mGy). Furthermore, in another study, Cui and Tang [19] reported that the calibration coefficient of GR-200 is different when it is received the various doses, for instance, the calibration coefficient is 0.05 and 0.021 for dose of 0.1 mGy and 20 mGy, respectively.

**Table 2:** Uncertainty components of TLDs

Factor	Uncertainty values (%)	

	TLD-100	GR-200
Calibration coefficient (N)	0.46	0.6
Fading correction factor (F_fad_)	0.02	0.05
Holder correction factor (F_hol_)	0.3	1
Energy correction (F_energy_)	1.10	2.3
Dose response non-linearity correction factor (F_lin_)	0.90	1
Uc	1.55	2.76

TLD: Thermoluminescent dosimeters

The estimated uncertainty of the TLDs from repetitive measurements at different distances was about 1.55% for TLD-100 and 2.76% for GR-200 (Table 2) which is in close agreement with a previous study [20].

### 3.3. TLD measurements and TPS calculations

**Figure 3:**
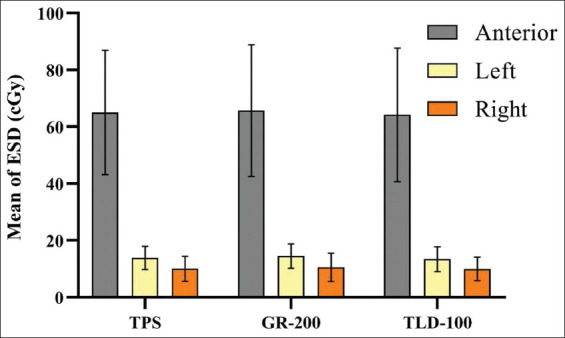
Mean of skin dose value (cGy) of treatment planning system and thermoluminescent dosimeters at three points

**Figure 4:**
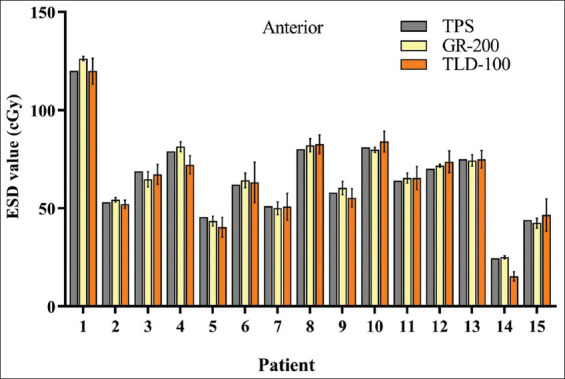
Calculated dose of treatment planning system and thermoluminescent dosimeters at the “anterior” point for each patient

**Figure 5:**
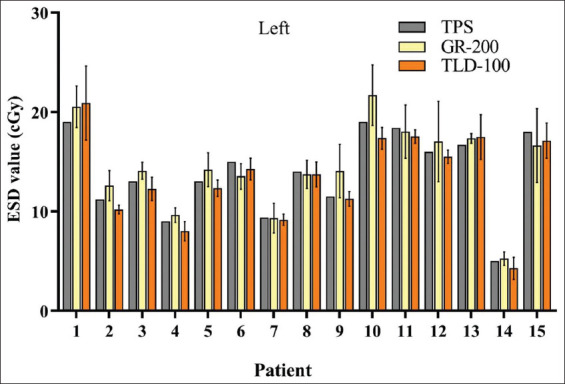
Calculated dose of treatment planning system and thermoluminescent dosimeters at the “left” point for each patient

**Figure 6:**
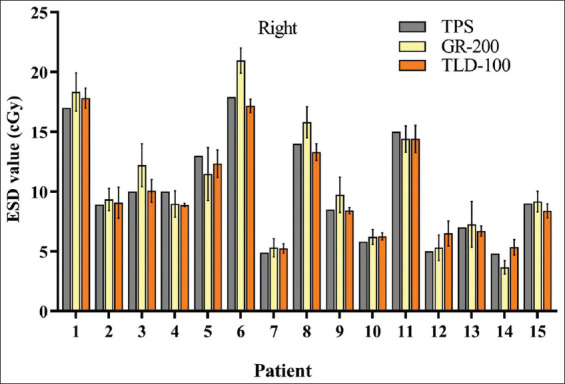
Calculated dose of treatment planning system and thermoluminescent dosimeters at the “right” point for each patient

Figure 3 represents a comparison between the mean skin dose value obtained from TPS, TLD-100, and GR-200. Furthermore, for each patient, the skin dose values at the anterior, left, and right points are reported in Figures 4-6. Due the high error probability in the posterior part, the dosimeter was not set out in this part. The skin dose depends on the prescribed dose, tissue inhomogeneity, and patient’s body mass index. Higher prescribed dose and thinner patient lead to greater skin dose.

Considering the TLDs uncertainty which was <2.8% for both dosimeters, approximately 70% of all cases showed a good agreement between TPS dose calculations and TLDs measurements. Raffi *et al*. [16] investigated the skin dose value using Monte Carlo simulations and TLD dosimeter for HDR ^192^Ir source in 35 patients. They reported that there was a good agreement (2% variation) between these measurements. They expressed that the TLD dosimeters have the potential to evaluate the skin dose value when the proper corrections are applied.

Following the treatment planning guideline, tissue dose (delivered dose) should not differ more than ~5.5% with TPS dose calculations [21]. Thereby, the difference (%) of three points in each patient is calculated and illustrated in Table 3. The positive values show that the TLD dose measurement were higher than the TPS calculated doses and the negative values imply the opposite. As shown in Table 3, TLDs reading doses at the anterior points have less disparity compared to the TPS dose predictions (deviation <5%), because in these points, the distances of TLDs from the sources are lesser than the lateral (left and right) points. According the general guidelines in treatment planning of radiation therapy, the prescribed dose in brachytherapy should not differ by more than 5% from the measured values [22]. Furthermore, the results demonstrated that TLD-100 has lower difference with TPS calculation in comparison to GR-200.

**Table 3:** Differences (%) between TLD-100 and GR-200 measurements with respect to TPS-calculated

Patient number	GR-200			TLD-100		
	
	Anterior	Left	Right	Anterior	Left	Right
1	3.84	4.08	4.39	1.12	6.58	3.3
2	1.62	3.48	2.13	−1.25	−2.1	1.73
3	−2.69	3.58	7.82	−1.54	−2	2.53
4	2.14	3	−3.17	−3.55	−4.09	−3.92
5	−3.91	4.13	−6.29	−4.71	−2.25	−2.36
6	2.06	−3.15	8.59	1.58	−2.88	−2.96
7	−1.75	−1.31	2.7	−1.01	−1.34	2.24
8	1.98	−1.38	4.75	3.03	−1.61	−2.84
9	2.35	7.34	3.36	−2.3	−3.99	−1.18
10	−1.27	8.49	2.02	2.33	−4.07	2.38
11	1.53	−2.13	−2.67	1.32	−3.28	−3.07
12	1.73	2.3	1.59	2.78	−2.18	4.59
13	−1.9	2.45	1.74	1.19	3.86	−1.32
14	1.82	1.56	−3.78	−7.4	−2.23	1.24
15	−2.63	−2.95	1.3	3.19	−3.33	−4.54

TLD: Thermoluminescent dosimeters, TPS: Treatment planning system

TLD measurements for “patients 14” showed a higher skin dose about 20% in the right position for GR-200, and also anterior (37%) and left (22%) positions for TLD-100 compared to the TPS calculations. In addition, in “patient 2” the variation of skin doses was more remarkable in the left position for both TLDs compared to the TPS calculations. The movements of markers on the patient’s skin during the CT-imaging and treatment rooms can be the main reason. Regarded to the previous study, 35% of point dose difference are due to 1.2 cm displacement of tandem in caudal-cranial direction [23]. Using portal imaging during brachytherapy would suppress the displacement.

Although TLD dosimeters are incapable to be used in real-time measurements, its lower price than other dosimeters, for instance MOSFET and diodes; hence, we used them as a preliminary assessment in clinical tests.

As future research, it is suggested that image-guided technique should be applied to find a correlation between skin dose and organ motion or applicator movements during HDR brachytherapy method.

## 4. Conclusion

In this study, skin dose of 15 patients during the brachytherapy of prostate malignancy was investigated. The skin dose measured by TLD-100 and GR-200 and compared with TPS calculation. The results indicated that there is a good agreement (about 70%) between the TLD measurements and TPS calculations with lower differences of TLD-100 in comparison to GR-200.
